# Women with pelvic organ prolapse and fibulin-5 rs12589592 polymorphism

**DOI:** 10.1590/1806-9282.20240687

**Published:** 2024-12-02

**Authors:** Fabiana Garcia Reis Maeda, Claudia Cristina Palos, Cesar Eduardo Fernandes, Ricardo Peres do Souto, Emerson de Oliveira

**Affiliations:** 1 Faculty of Medicine of ABC, Molecular Biology Laboratory – Santo André (SP), Brazil.; 2 Faculty of Medicine of ABC, Department of Gynecology and Obstetrics – Santo André (SP), Brazil.; 3 Faculty of Medicine of ABC, Department of Biochemistry – Santo André (SP), Brazil.

**Keywords:** Pelvic organ prolapse, Case-control studies, Single nucleotide polymorphism, Restriction fragment length polymorphism

## Abstract

**OBJECTIVE:**

This study aims to access the frequency of single-nucleotide polymorphism rs12589592 (G>A) of the fibulin-5 gene in a Brazilian population with pelvic organ prolapse.

**METHODS:**

This was a case-control study, with menopausal women divided into two groups and classified using the pelvic organ prolapse quantification system: pelvic organ prolapse group: pelvic organ prolapse quantification system stages III and IV and Control group: pelvic organ prolapse quantification system stages I and 0. We collected epidemiologic and baseline health information and performed genotyping of rs12589592 from the fibulin-5 gene using a restriction fragment length polymorphism (polymerase chain reaction-restriction fragment length polymorphism) strategy, based on the distinction of sequences from alleles G and A by the restriction enzyme *Dde*I. For the pelvic organ prolapse group and control, 111 and 180 women were recruited, respectively.

**RESULTS:**

The rs12589592 (G>A) polymorphism analysis showed 141 GG homozygotes (pelvic organ prolapse group: 53 [47.7%] and Control: 88 [49.2%] p=0.850); and 149 AA+GA: (pelvic organ prolapse group: 58 [52.3%]; Control: 91 [50.8%]; p=0.904). The distribution of genotypes did not follow the Hardy-Weinberg equilibrium conditions.

**CONCLUSION:**

There was no difference between groups regarding genotypes (rs12589592 G>A) frequency; however, the population characteristics prevent the analysis of the association between the genotype and the occurrence of prolapse.

## INTRODUCTION

Pelvic organ prolapse (POP) is a highly prevalent condition that can be detected in up to 50% of women in vaginal examination^1^. Its symptoms may require surgical treatment, with a 12.6% risk of having a POP correction surgery until the age of 80^2^. Recent insights emerged from studies with mice addressing the primary role of disordered elastic fiber homeostasis in the pathogenesis of pelvic organ prolapse^3^. So far, three genes whose deletion in mice causes POP have been identified: lysyl oxidase-like-1, fibulin-5, and fibulin-3^3-5^.

Fibulin-5 is a critical molecule for elastic fiber assembly, binding directly to tropoelastin and regulating protease activities in an integrin-dependent manner^6^. These molecular modifications may interfere both in the formation of elastic fiber of the pelvic floor during embryogenesis and in the remodeling of elastic fiber in pregnancy and childbirth^7^. The fibulin-5 gene (*FBLN5*) knockout model shows 92% of prolapse in female mice after 6 months of life^3^.

Fibulin-5 dysregulation has been confirmed in tissues from humans with POP by several studies showing its lower gene and protein expression in vaginal walls and in cardinal and uterosacral ligaments^8-13^. Researchers went forward to uncover the genetic variability of the *FBLN5* gene in women with POP^14^. Eleven of the most common single-nucleotide polymorphisms (SNPs) located near this gene in humans were studied, and significant association with POP was found for two of them: rs2018736 and rs12589592^14^.

The genetic aspects, including polymorphisms, related to POP in a Brazilian population have been previously explored^15-19^. This study adds up to a database with distinct characteristics from the ones already available, particularly on ethnical aspects, collaborating to early POP diagnosis, treatment, and prevention. The study objective is to determine the frequency of variants from SNP rs12589592 in the fibulin-5 gene in a Brazilian population and its association with POP.

## STUDY DESIGN

This was a case-control study conducted among women assisted by the Urogynecology and Pelvic Floor Dysfunction Sector of the Department of Obstetrics and Gynecology at a tertiary center to evaluate the frequency and association between pelvic organ prolapse and one *FBLN5* gene polymorphism.

### Ethical aspects

The study was approved by the institutional ethics (CAAE 27382014.1.0000.0082) and followed the international and local guidelines for ethical practices in research with humans. All participants were informed about the study and signed the Informed Consent Form.

### Patients

All consecutive women assisted by the Urogynecology and Pelvic Floor Dysfunction Clinic during the period of 2014–2016 without menstrual flow for at least 1 year, no previous vaginal surgery (except episiotomy), and without hormone therapy for at least 6 months were included. Women with neoplasia or with connective tissue disease were excluded from the study. Quantification of prolapse was performed in gynecological examination by the researchers, using pelvic organ prolapse quantification (POP-Q) staging, following the recommended classification of the International Continence Society, American Urogynecology Society, and Society of Gynecologic Surgeons^20^. Patients with pelvic organ prolapse stage III or IV were included in the POP group. In the Control group, women without prolapse (stage 0) or minimal prolapse (stage I) were selected. The sample size was 112 cases and 180 controls. Information from patients, including name, hospital registration number, age, obstetric history, and age of menopause, was retrieved from medical records and anamnesis. Weight and height data were collected by the researchers. Data from this cohort have also been analyzed in other studies for different outcomes^15-19^.

### Genetic analysis

Polymorphism rs12589592 is a G>A variation located in one intronic region of the *FBLN5* gene. A novel restriction fragment length polymorphism (RFLP) strategy for genotyping this SNP was developed based on the *FBLN5* gene sequence available from GenBank (NC_000014.9). Primers for amplification of the region containing rs12589592 were chosen using the Primer3 program^21^. The WatCut tool, available from the University of Waterloo at http://watcut.uwaterloo.ca/template.php, suggested the restriction enzyme *Dde*I to discriminate G and A gene variants.

Genomic DNA from participants was obtained from blood collected with EDTA using the illustra blood genomicPrep Mini spin kit (GE Healthcare). About 100 ng of extracted DNA was amplified by polymerase chain reaction (PCR) in a 20 μL reaction using PCR Master Mix (Promega) and 0.5 μM of primers 5′-CCTTCGTTTCCTCGTTTGTG-3′ (sense) and 5′-TTTCCTTTTGCATCCTCCAG-3′ (anti-sense) in a Mastercycler Personal thermocycler (Eppendorf). The temperature protocol for amplification was: initial step of 5 min at 94°C, 45 cycles of three temperatures (30 s at 94°C, 60 s at 52°C, 60 s at 72°C), and closing step of 10 min at 72° in the amplified *FBLN5* gene region is 459 bp in size. To determine the alleles, the PCR product was treated with 1U *Dde*I (Thermo Fisher Scientific) for 3 h at 37°C. In DNA amplified from the A allele, there are two *Dde*I sites, yielding three fragments after digestion: 297, 135, and 27 bp products. In the PCR product from the G allele, there are three *Dde*I sites, yielding four fragments: 178, 135, 119, and 27 bp. Restriction products were separated and analyzed by 2.5% agarose gel electrophoresis. In this type of gel, the 27 bp fragment is not visualized, and the 135 and 119 bp fragments run remarkably close. Genotyping is made by analyzing the 297 and 178 bp bands: only the former is present in AA homozygotes, only the latter is present in GG homozygotes, and both are present in AG heterozygotes.

### Statistical analysis

The normality of quantitative data was verified with the Shapiro-Wilk test. The chi-square test and Fisher's exact test were used to compare qualitative variables. To analyze the quantitative variables, the Student's t-test was used. The deviation from Hardy-Weinberg equilibrium was calculated by the chi-square test. The value of statistical significance was set at 5%. Analyses were performed using GraphPad Prism 6 or SPSS version 23.

## RESULTS

**Figure 1:**
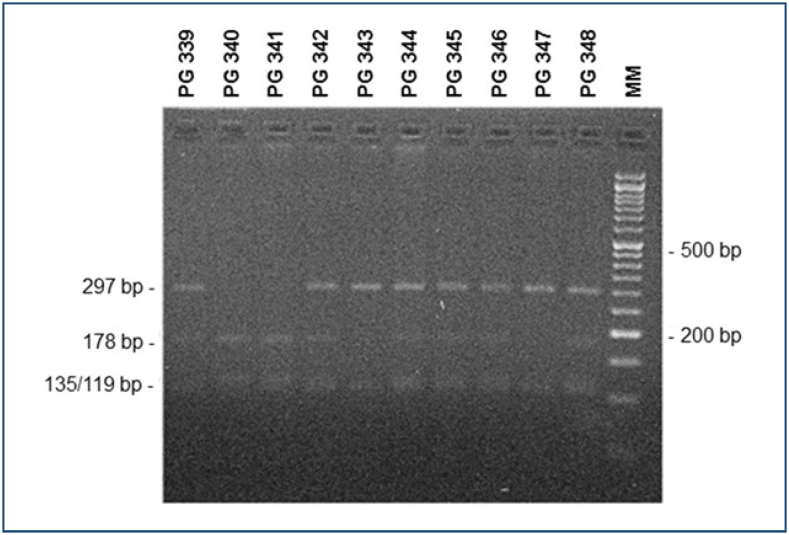
Picture of the genotyping instrument used in the study. Representative gel of polymerase chain reaction-restriction fragment length polymorphism analysis: 2.5% agarose gel electrophoresis of *Dde*I digested polymerase chain reaction fragments for genotyping SNP rs12589592 of the *FBLN5* gene. Homozygous GG: patients PG340 and 341; Heterozygote AG: patients 339, 342, 344, 345, 346, and 348; AA homozygote: patients 343 and 347. MM: molecular marker (50 bp DNA ladder).

At the end of recruitment, 292 women were included. The POP group had 112 women, and the Control group had 180 women. The mean age of the Control group was 57.8 years and the POP group was 68.4 years old; the median number of pregnancies was 3 for the Control and 4 for the POP groups. Each group lost one member due to the impossibility of obtaining a genotype for the rs12589592 polymorphism. The groups were heterogeneous concerning age, parity, number of pregnancies, number of vaginal deliveries, and home deliveries (Table 1). The genotyping method example is shown in Figure 1. The rs12589592 polymorphism analysis showed 141 GG homozygotes, 81 GA heterozygotes, and 68 AA homozygotes (Table 2). This population did not follow the Hardy-Weinberg equilibrium conditions. The frequencies of alleles G and A observed in this population were 0.63 and 0.37, respectively. Regarding POP, there was no difference in the distribution of genotypes between the case and control groups, considering both the three genotypes separately and combining the less frequent allele homozygotes with heterozygotes.

**Table 1:** Baseline characteristics.

Variables	Group control (n=180)	Group POP (n=112)	p-value
	Mean. median or %	Mean. median or %	
Age	57.8	68.4	<0.0001
Ethnicity white	64.8%	69.9%	0.42
Non-white	35.2%	30.1%	
Body mass index (kg/m^2^)	28.9	28.8	0.87
Age at menopause	46.6	48.8	0.07
Hormonal therapy	18.1%	10.7%	0.09
Smoking	20.1%	13.1%	0.15
Hypertension	49.4%	57.8%	0.18
Diabetes mellitus	23.7%	24.5%	0.88
Dyslipidemia	24.7%	25.4%	0.88
Chronic cough	6.8%	1.8%	0.08
Constipation	10.4%	14.3%	0.35
Pregnancies	3 (0–16)	4 (1–24)	<0.0001
Parity	3 (0–15)	4 (1–16)	<0.0001
Vaginal delivery	2 (0–15)	3 (0–16)	<0.0001
Cesarean	0 (0–3)	0 (0–1)	0.37
Highest newborn weight (g)	3,059	3,516	0.14
Episiotomy	9.2%	8.3%	>0.99
Labor analgesia	4.8%	3.7%	0.76
Home birth	3.05%	25.9%	<0.0001
Hysterectomy	15.6%	15.2%	>0.99
Activities with exaggerated physical exercise	14.1%	22.5%	0.07

1 Group control: women with stage 0 or I pelvic organ prolapse. Group POP: women with stage III or IV pelvic organ prolapse. Statistical test for values expressed as the mean: Student's t-test. Statistical test for values expressed as percentages: Fisher's exact test. POP: pelvic organ prolapse.

**Table 2:** Distribution of pelvic organ prolapse case genotypes (pelvic organ prolapse) and controls for single-nucleotide polymorphism rs12589592 of the *FBLN5* gene.

Genotypes	POP (n=111)	Controls (n=179)	p
GG	53 (47.7%)	88 (49.2%)	0.850*
GA	30 (27.1%)	51 (28.5%)	
AA	28 (25.2%)	40 (22.3%)	
GG	53 (47.7%)	88 (49.2%)	0.904**
GA+AA	58 (52.3%)	91 (50.8%)	

* Chi-square test;

** Fisher exact test. POP: pelvic organ prolapse.

## DISCUSSION

In this study, 112 women with advanced prolapse were compared to 180 women with minimal or without prolapse. Using a novel PCR-RFLP strategy, the genotype of the rs12589592 polymorphism in the *FBLN5* gene was determined. No significant difference was observed between the distributions of SNP genotype in groups, considering either three individual genotypes or combining heterozygotes and AA homozygotes. Our result differs from others who found a lower frequency of AA among affected women and risk effect of this genotype for POP^14,22^.

The analysis of candidate genes in different regions, populations, and races is often inconsistent. In 2014, Khadzhieva et al.^14^ studied a Caucasian population in Russia, including pre- and post-menopause women, and obtained case and control statistically distinct groups regarding age, vaginal parity, and perineal trauma in childbirth. This stresses the difficulty that we also faced in obtaining comparable groups once the risk factors are so determinant for POP occurrence. The effect size of having a lower specific allele frequency than controls was only relevant to the groups with perineal trauma at birth or macrosomic new born.

Abulaizi et al., in 2020, studied a cohort of a particular ethnicity in China and also found a lower frequency of the AA genotype in the POP group, among other SNPs^22^. Both research studies are extremely specific for their study population; however, added to our data, they may contribute to a wider panorama where diversity can play a role in genetic determinants for POP.

In our study, the frequency for the less common allele of rs12589592 was 0.37. This value is within the range reported in the dbSNP (single-nucleotide polymorphism database) for this polymorphism (https://www.ncbi.nlm.nih.gov/snp/rs12589592). In our sample, the distribution of rs12589592 polymorphism genotypes calculated is not in Hardy-Weinberg equilibrium. Some issues could justify this deviation, including biological phenomena and technical problems.

There is no reason to imagine that any rs12589592 allele represents a selective disadvantage since this polymorphism was in equilibrium in another study^14^. Regarding technical aspects, errors in genotyping and questions in sampling could be considered. Indeed, there is concern about the accuracy of PCR-RFLP in determining SNP variants^23^. For ratification of the results, all genotypes were confirmed by a second PCR-RFLP strategy with distinct primers followed by *Dde*I digestion (data not shown). Thus, the inability to fit the Hardy-Weinberg model could be related to particularities of this sample.

The gene signal for the production of ﬁbulin-5 is decreased in the supportive extracellular matrix in women with POP^9^. The present study did not address the gene expression. The association between FBLN5 polymorphisms and gene expression has not yet been investigated and should be the subject of further studies on genetic susceptibility to POP.

The lack of difference between our study groups regarding rs12589592 polymorphism may further contribute to the debate about the importance of genetic aspects to the onset of POP. Even among the most promising polymorphisms for POP risk factors, such as those located in the COL1A1 and COL3A1 collagen genes, there is no consensus in studies on the association between genetic variants and prolapse^24,25^. Those discrepancies may be attributed to genetic differences between populations and particularities of the experimental design of each study.

This study has limitations regarding sample size and genotyping methodology. Although the sample is comparable to other studies evaluating POP gene polymorphisms^15,16,18^, it may still be small to detect more subtle differences. Regarding the determination of genotypes by PCR-RFLP, this is a traditional and well-established procedure, but with inferior results to DNA sequencing, the gold standard of nucleotide sequence analysis.

There was no difference between groups regarding genotype frequency, suggesting a lack of association between rs12589592 of the fibulin-5 gene and POP in this particular population; however, the interpretation of the association is impaired due to the lack of Hardy-Weinberg equilibrium conditions.

## ETHICAL APPROVAL

All procedures followed were in accordance with the ethical standards of the responsible committee on human experimentation (institutional and national) and with the Helsinki Declaration of 1964 and its later amendments. The study was evaluated by the institutional ethics committee (CAAE 27382014.1.0000.0082) and approved on 03/26/2014 (Process # 554.670).

## INFORMED CONSENT

All participants were informed about the study and signed the Informed Consent Form.
